# Health technology assessments as a mechanism for increased value for money: recommendations to the Global Fund

**DOI:** 10.1186/1744-8603-9-35

**Published:** 2013-08-21

**Authors:** Yot Teerawattananon, Kate McQueston, Amanda Glassman, Jomkwan Yothasamut, Chaw Yin Myint

**Affiliations:** 1 Health Intervention and Technology Assessment Program (HITAP), Department of Health, Ministry of Public Health, 6th floor, 6th Building, Tiwanon Rd., Nonthaburi 11000, Thailand; 2 Center for Global Development (CGD), 1800 Massachusetts Avenue NW, Third Floor, Washington, DC 20036, USA

**Keywords:** Global Fund, Health technology assessment, Cost-effectiveness analysis, Program evaluation, Global health

## Abstract

The Global Fund is experiencing increased pressure to optimize results and improve its impact per dollar spent. It is also in transition from a provider of emergency funding, to a long-term, sustainable financing mechanism. This paper assesses the efficacy of current Global Fund investment and examines how health technology assessments (HTAs) can be used to provide guidance on the relative priority of health interventions currently subsidized by the Global Fund. In addition, this paper identifies areas where the application of HTAs can exert the greatest impact and proposes ways in which this tool could be incorporated, as a routine component, into application, decision, implementation, and monitoring and evaluation processes. Finally, it addresses the challenges facing the Global Fund in realizing the full potential of HTAs.

## Introduction

The Global Fund, created in 2001 as a global financing mechanism, enables low-income countries (LICs) and middle income countries (MICs) to promote access to certain health interventions and technology for the prevention and treatment of HIV/AIDS, tuberculosis, and malaria. Given the commitment from its donors—amounting to almost $30.5 billion in pledges and $24 billion in contributions to date—as well as its scale of work in approximately 100 countries [1], the Global Fund has emerged as one of the most significant global health players over the last decade. Its total disbursements in 2009 constituted 3.29% of total health expenditure in LICs, 0.22% in low MICs and 0.07% in high MICs, while its contribution to individual countries ranged from 0.002% in Botswana to 53.4% in the Democratic Republic of Congo [2].

As a global health financier rather than a technical or implementing institution, the Global Fund does not operate directly within countries or implement its own programs [3]. Historically, the Global Fund has issued “calls for proposals” for applications through a rounds-based mechanism. As of August 2012, there have been 10rounds. At the time of writing, this system is in the process of being replaced by a funding model allowing for more flexible timing of grant applications. Global Fund affiliated partnerships—termed Country Coordinating Mechanisms (CCMs)—are tasked with developing proposals based on stakeholder consultations, local funding needs, and epidemiological context. The Global Fund notes that the applicant is responsible for “deciding their own priorities, strategies and programs.” Proposals are submitted to the Secretariat, verified for eligibility, and reviewed for completeness by a Technical Review Panel (TRP), which in turn makes funding recommendations to the Global Fund Board. The TRP consists of representatives with an array of expertise, both scientific and programmatic, as well as program experience in HIV/AIDS, tuberculosis and/or malaria. The TRP terms of reference direct the panel to review grant applications against technical criteria including feasibility, value for money, and sustainability [3].

If a proposal is approved, funds can be disbursed under the supervision of the CCM to the Principal Recipients (PRs) and/or Sub-Recipient(s) (SRs) who are responsible for the program’s implementation. In addition, a Local Fund Agent (LFA), an independent body contracted by the Global Fund Secretariat, is responsible for monitoring the PR’s performance, grant implementation, and financial reports. Global Fund grants can be made for a period of up to five years, although funds are typically reviewed after two years, with continued funding conditional upon performance [3] and other factors [4].

As donor funding for global health declines due to the global recession, the Global Fund is experiencing increased pressure to optimize results and improve impact per dollar spent [1]. In its recent strategic plan [5] covering the 2012–2016 period, the Global Fund sets ambitious goals to increase impact by investing strategically in areas with high potential and offering strong value for money. Despite its clear objectives on “maximizing impact and value for money,” there are still major challenges in the implementation of the plan. This paper reviews the current successes and impediments of Global Fund investment and examines how health technology assessment (HTA) can be used to provide guidance on the relative priority of health interventions (medications, devices, diagnostics, and other treatment modalities) subsidized by the Global Fund. In addition, this paper aims to identify areas where the application of HTA could have the greatest impact and to propose ways in which it could be incorporated, as a routine component, into application, decision, implementation, and monitoring and evaluation processes. Finally, it addresses the challenges facing the Global Fund in realizing the full potential of HTA.

### The improved performance, transparency, and efficiency of the Global Fund

As of 2013, the Global Fund has disbursed a total of over $20 billion in 151 countries [6]. Its investments have likely contributed to the significant increase in the number of HIV/AIDS patients receiving antiretroviral treatment (from an estimated 300,000 in 2002to 5.25 million by 2009 [7]) as well as the number of insecticide-treated nets (ITNs) distributed in 35 high-burden African countries (from an estimated 10 million in 2004 to 35-44million per year between 2006 and 2008 [5]), as well as the detection rate of new smear-positive tuberculosis cases (from 36%– 44% in 2000 to 55%–67% in 2008 [8]).

The Global Fund is governed in a unique way, both at a board and implementation level. Leadership is sourced from within developing counties, the private-for-profit sector, and civil society. There is a strong commitment to increasing transparency and accountability, and as a result, the fund has improved the availability and accuracy of information related to the disbursement of funding and coverage of specific services, including the establishment of specific surveillance of the focal diseases, despite difficulties involving duplication of information systems in some recipient countries [7]. In addition, the Global Fund has pursued the principle of performance-based funding that is disbursement of funds has been largely correlated with grant performance; for example, the best performing programs receiving 79% of their grant sums compared to 38% for the worst performers [9].

Although there is an argument that performance-based funding systems might place LICs at a disadvantage due to their comparatively poor access to resources and capacity, at least three studies have rejected this hypothesis [2,10,11]. These studies reveal that when taking other factors into consideration, grants in LICs have tended to out-perform their more resource-rich counterparts. Lu and colleagues [10] reported that an increase in per-capita income from $1000 to $2000 is associated with a substantial reduction in disbursement of 2-year grant sums (an indicator of both expenditure and performance, in view of the Global Fund’s incremental disbursement system). Radelet and Siddiqi [11] demonstrated a similarly strong negative relation between the income and achievement of programmatic targets. The authors concluded that poor nations, including so-called fragile states, had proven themselves capable of effectively utilizing increased funding flows from the Global Fund. Moreover, the most important finding is a significant negative association between grant implementation rates and income per person, suggesting that LICs are more likely to disburse grants from the Global Fund than countries with higher per capita income.

Although the Global Fund has dedicated itself to increasing prevention and control of AIDS, tuberculosis, and malaria, it has also made significant investments in improving the health systems of LICs [12]. Flexibility of the financial support from the Global Fund allows recipient countries to strengthen their health systems through a number of approaches, ranging from health worker training sessions and salary support to improved workforce retention and electronic health records systems. Such efforts may not only facilitate the success of AIDS, tuberculosis, and malaria programs, but also ensure that scarce health system resources are not diverted to these three diseases at the expense of other health needs. In 2009, 35% of its funding contributed directly to supporting human resources, infrastructure and equipment, and monitoring and evaluation in health systems [13]. AIDS treatment programs themselves benefit from investment in health systems, as healthcare workers benefit directly from improved systems and increased access to antiretroviral treatment results in fewer patient admissions to hospital, which then helps free up health workers and related resources to that they can be devoted to other health needs [13].

### The impediments and challenges

As with a number of global health initiatives, including the GAVI Alliance, the policies and priorities of the Global Fund are defined at a global level. Although the Global Fund works to maintain relatively high levels of national ownership in its programming, evidence suggests that there remains misalignment between its policies and programs and those of national governments. As a result, services that are managed both by the government and the Global Fund are often badly coordinated and inefficiently managed, with duplication of tasks—including reporting, monitoring and evaluation, and funding/disbursement mechanisms—representing significant obstacles to efficiency [12,14]. Delays in disbursement of funds to the PRs and donor short falls in financial pledges have both emerged as significant challenges [1]. The significant fall in donor funding in the 2011–2013 funding round led directly to the cancelation of the eleventh call for proposals. As a result of these issues, efficiency improvement has been defined as a central tenet of the Global Fund strategic plan for 2012–2016.

Several studies have identified efficiency shortfalls within the Global Fund. At a macro-level, Zhao et al. [15] reviewed performance indicators for Global Fund malaria programs and identified an over reliance on input indicators—especially those related to training activities—at the expense of outcome or impact indicators, which are better suited to measuring disease reduction. This tendency to set inappropriate indicators may distort performance ratings and, consequently, grant funding [15]. This has been seen in Timor-Leste, where effective strategies for controlling malaria receive less funding than behavioral change activities, despite the fact the former approach has been found to be more effective in disease prevention [16]. For instance, both ITN distribution programs (which have been found to be very effective in preventing malaria in high transmission areas [17]) and case management (improved diagnosis), another highly effective intervention, were clearly underfinanced, receiving less than 1% of the total grant support [16], in favor of behavioral change programs.

This situation is repeated across HIV and tuberculosis programs. Effective and efficient prevention and control depend on implementing the right mix of interventions for each setting and assuring the necessary coverage of those interventions. Bridge et al. [18] found that less than half HIV proposals funded by the Global Fund included harm reduction activities, even though many studies confirm that these activities offer good value for money [19,20]. Moreover, although there is strong evidence that male circumcision can reduce HIV transmission in men by up to 60% [21], we found that no Global Fund proposals included circumcision initiatives. At the same time, many interventions that aim to influence knowledge, attitudes, and beliefs and influence psychological and social correlates of risk have received significant support from the Global Fund [22], despite the fact that the impact of these programs—including whether they bring about sustained long-term behavior change—remains uncertain [23]. Korenromp et al. [24] suggest that the Global Fund could significantly enhance the impact of its tuberculosis investment by reevaluating its investment across regions, for instance by prioritizing investment in Africa, and by screening and treating tuberculosis in populations with high levels of HIV infection. A recent report developed by the Value for Money Working Group (2013) reaffirmed the limitations of the Global Fund’s investments in the TB, HIV/AIDS and Malaria programs and it also provided suggestions to improve funding strategies.

Aside from the issue of effectiveness, an increasing amount of information on resource use and health consequences, i.e. cost-effectiveness, has been amassed in recent years. Global health professionals no longer focus only on effective prevention and treatment; instead, more sophisticated models that examine cost-effectiveness and comparative effectiveness as a way of improving public health without requiring significantly more funding are at the centre of most public health initiatives. The differences in cost-effectiveness between interventions can be staggering, particularly when considering initiatives that require implementation at scale. For instance, despite massive increases in access to HIV treatment, WHO and UNAIDS estimate that, as of 2011,there are still as many as 15 million people around the world in need of antiretroviral therapy. To treat populations of this scale, the difference in cost-effectiveness between the most cost-effective treatment option and the least can be as much as 1,400 fold (Disease Control Priorities in Developing Countries Project) [25]. Even differences in intervention implementation can result in significant variation. A 2011 paper by Amole et al. [26] found that some Global Fund recipient countries are not currently optimizing their HIV treatment by selecting the most cost-effective antiretroviral regimen and implementation strategy (i.e. treating population who is most likely to further transmit HIV infection to other populations). Adjusting the current make-up of antiretroviral drug purchases in sub-Saharan Africa and India could yield over $300 million in savings over the next five years and expand the provision of quality services in resource limited settings.

Despite the Global Fund’s explicit commitment to implementing cost-effective and proven initiatives, it is clearly failing to fund programs that fulfill these criteria to the extent that it should. This may be because the Global Fund provides support to countries based on requests received from CCMs, who themselves may not have a clear idea about a country’s needs or the most cost-effective strategies that can be implemented to meet those needs. As a funding organization, the Global Fund has little in-house technical capacity and little direct engagement with countries to which they provide funding. There is no systematic support to PRs on whether proposed interventions are among the most effective and cost-efficient for achieving the desired outcome in a given context. Although the TRP has been set up to provide funding recommendations to the Board of the Global Fund for making final decisions, it is difficult for the TRP to assess technical soundness and value for money or to make rational recommendations on strategic investments based only on the data presented in the applications, especially when the information is weak, patchy, or inconsistent, and where funding is limited to specific time periods. Moreover, an absence of guideline on what information should be used in order to assess value for money and a lack of HTA capacity of the TRP secretariat prohibit the use of value for money information in TRP review process.

Furthermore, it is widely accepted that the impact and cost-effectiveness of interventions depends to a large extent on the strength of the health system within which they are delivered. A lack of absorptive capacity at all levels of grant implementation has been identified in many settings and may explain the slow progress in grant implementation outside of a robust health system [16,27]. Previous research on this subject reveals that inadequate institutional capacity and high staff turn-over negatively impacts organizational capacity, which can lead to poor performance in both project implementation and monitoring and evaluation [28-33]. Therefore, the Global Fund needs to ensure that relevant infrastructure, e.g. laboratory for HIV and CD4 test, is built before investment in commodities, e.g. antiretroviral drugs, are made. On the other hand, HTA focus on effectiveness (real-work effect) of investment rather than efficacy (potential effect in idea situation) so that weakness at system level can be taken into account appropriately in decision making process.

The situation can also be applied to the substantial investments in health information systems for improving healthcare services and enhancing management, monitoring and evaluation of the fund itself. Until now, very few studies have addressed this issue [34,35]. As a result, HTA on health information systems should be one of priority areas given that there is very little evidence of a comprehensive plan by the Global Fund in this area or attempts to standardize on a small number of well-established systems or to initiate any evaluations of such systems.

### Potential applications of HTA within the Global Fund

As is made clear in The Global Fund strategy 2012–2016, the organization must make the transition from an emergency funder to a long-term, sustainable financing mechanism. To this end, it needs to develop new risk-management approaches, strengthen internal governance, institute a new grant-approval process, strengthen decision making by middle management, and improve its focus on results [5]. Although this report does not provide direction on certain critical issues that will define the future success and impact of the Global Fund, it suggests that the Global Fund could achieve better value for money with better technical evaluation and management.

A 2011 report [36] by the Results for Development Institute on behalf of the Global Fund’s Market Dynamics Committee makes several recommendations for the optimization of product selection, including that the Global Fund commission global value for money guidance on specific products: “*An experienced independent expert body such as the National Institute for Health and Clinical Excellence (NICE) could be commissioned to conduct robust comparative cost-effectiveness analyses of two or more WHO-recommended products and provide that information to the Global Fund and its recipients.”* This is not the first time that academics have urged the Global Fund to consider the use of HTA to improve its cost-effectiveness [25]. The application of an HTA to a health initiative is a multidisciplinary activity that systematically examines the costs and benefits as well as the organizational implications and social consequences of the application of a health policy and/or technology. HTAs often function as a “bridges” between evidence and policy-making, providing health policy-makers with accessible, useable, and evidence-based information that can help guide their decisions regarding the appropriate use of technology [37].

HTAs not only generate a wide range of policy-relevant information that can aid decision making, but also empower stakeholders that are involved in the decision making process. This is because HTAs, as tools in a priority setting approach, are often designed according to a set of questions that themselves encourage a critical evaluation of the relevant social and financial factors [38]. This kind of evaluation can help decision makers unpack evidence and assess the relative importance of both process values (such as transparency, accountability, participation, legality, faithfulness to constitutional provisions, and respect for international obligations) and content values (such as clinical effectiveness, value for money, equity, solidarity, and feasibility). HTAs are particularly suitable for global organizations because they take into account the kinds of values that vary across settings as a result of differing social factors, including politics, culture, social demographics, religion, and levels of economic development.

**Figure 1:**
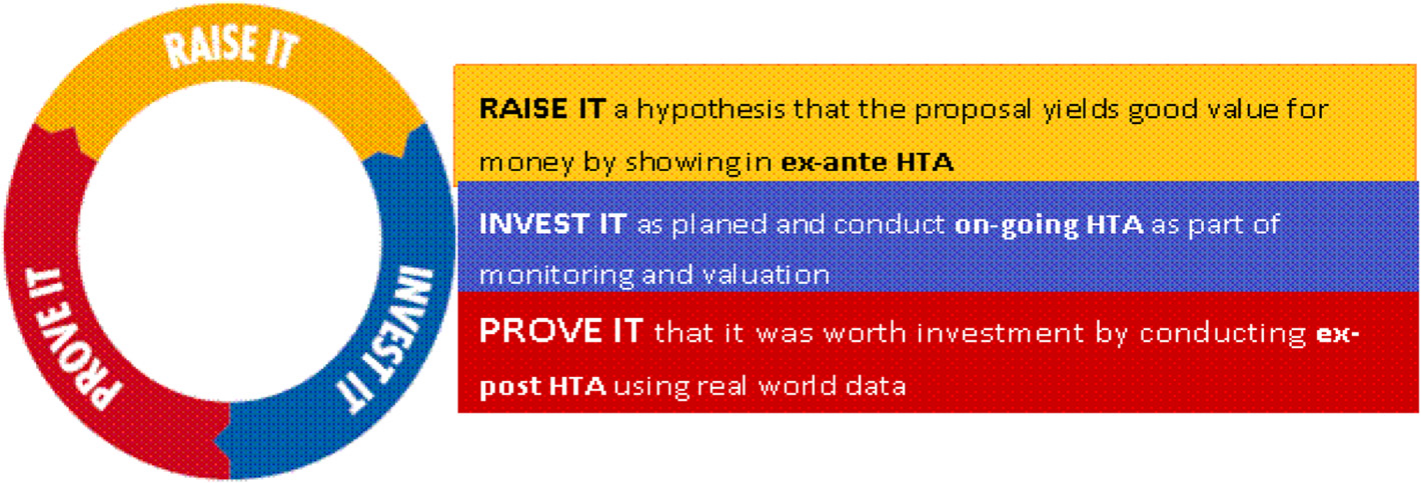
Potential use of Health Technology Assessment (HTA) to enhance value for money of Global Fund initiatives.

Figure 1 is a modified version of the Global Fund’s model of performance-based funding. It presents a method for enhancing the efficiency of Global Fund projects, through the use of HTA. HTAs can enhance value for money at all stages of the Global Fund process, from proposal development to final evaluation. For instance, with the inclusion of an HTA, proposals are far more likely to take into account cost and efficiency, among other factors, resulting in a much higher quality, rigorous, and evidence-based proposal. The higher the quality of the proposal submitted to the Global Fund, the more likely the donors will be to respond to the sustained level of demand for resources. In other words, the higher the quality of the proposal, the greater the impact obtained from investment. However, this task is not simple. Proposal development requires an increased focus on intervention or mixes of interventions that are locally appropriate, including assessments of affordability and cost-effectiveness in the given context. Cost-effectiveness information may be derived from the existing literature, including the Disease Control Priorities in Developing Countries project [17], as well as from the Global Fund’s value for money guidance—a collective set of comparable cost-effectiveness information of various interventions implemented in various settings. This supports the claim of Korenromp et al. [24] that proactive approaches from the Global Fund to inform demand on the kinds of initiatives that yield good value for money (and those that do not) would result in larger numbers of lives saved than might be the case with the prevailing funding model, which relies heavily on country demand.

We suggest not only that cost-effective interventions be selected correctly from *the value for money guidance*, but also that HTA be applied to at least some aspects of a proposal before implementation. These ex-ante HTAs would use existing evidence and assumptions to estimate the likely costs and impacts of the proposed program (Table 1). Having HTA as a pre-condition may well drive local data generation through countries’ initiative and also by the Global Fund. Relevant stakeholders should work with HTA experts to ensure the local relevancy of the assessment, as well as to strengthen the absorptive capacity of the grant recipients by facilitating the consideration of important parameters (factors) affecting the potential success of grant implementation. The results of these ex-ante HTA can then also be used as a baseline for conducting monitoring and evaluation once the program is implemented. An ex-ante assessment can be used as a requirement for a CCM that does not select the cost-effective interventions in its proposal to demonstrate that the selected intervention(s) is at least as cost-effective in a given context if not more so than those reported in *the value for money guidance*. It is also expected that this approach will help create shared priorities of the Global Fund and its recipients.

**Table 1:** Examples of ex-ante and ex-post health technology assessments

**A case study of ex-ante assessment of the feasibility and value for money of the maternal and child health voucher scheme in Myanmar **[39]	**A case study of an on-going HTA of HIV prevention for the most-at-risk population in Thailand **[40]	
An ex-ante assessment was conducted as part of a collaborative study undertaken by Myanmar’s Ministry of Health, WHO, and the Thai Ministry of Public Health between March 2010 and September 2011. The aim of the assessment was to collect information to guide the formulation and implementation of a demand-side financing mechanism for maternal and child health (MCH) services in Myanmar. The main objective of the MCH voucher scheme is to eliminate the financial barriers to maternal and child health care among poor households by providing support in the form of four antenatal visits, delivery by skilled birth attendants, postnatal care, transportation, food, and lodging. Using both qualitative and quantitative methods, including an economic evaluation, this collaborative research demonstrates that the use of demand-side financing for MCH services in Myanmar appears to be feasible and represents good value for money. The evidence suggested that the initiative was likely to garner support from community leaders and civic groups, and be accepted by target populations and health workers, because it removes many of the impediments that people currently Figure 1 Potential use of Health Technology Assessment (HTA) to enhance value for money of Global Fund initiatives. Teerawattananon et al. Globalization and Health 2013, 9:35 Page 5 of 9 http://www.globalizationandhealth.com/content/9/1/35 encounter when trying to access MCH services. Some of the most common barriers that people face when trying to access these services are the long distances between the residence of the mother and the nearest health facility, and the related high travelling costs (particularly in rural areas), the high cost of medicines (which for many is unaffordable). In Myanmar, where the average number of pregnancies per year is 900,000, it is estimated that introducing the MCH voucher scheme would increase ANC coverage from 68% to 93% and delivery by skilled-birth attendants from 50% to 71%. The ex-ante assessment found that the MCH voucher scheme was likely to save a significant number of lives of mothers and infants, for whom the cost of ANC is currently prohibitive. The assessment also found that this could be done at a reasonable cost. The incremental costeffectiveness ratio (ICER), which in this case is the additional cost per life-year saved from introducing the MCH voucher scheme compared to the status quo, ranged from 376,548 to 452,110 kyats (475 kyats = 1 international dollar, in 2010). This represents good value for money, especially given the ceiling threshold of 1 time of GDP per capita of 413,800 kyats. The results of this study were presented to senior decision makers in Myanmar in March 2011 resulting in an agreement being reached to implement the MCH voucher scheme in one township commencing in November 2012 before scaling it up as a nationwide program.	Global Fund for a (Round 8) grant support of $75.46 million over five years, from July 2009 to May 2014. The three principal recipients (PRs) are the Thai Ministry of Public Health and two non-governmental organizations. This program aims to expand HIV preventive services for female sex workers (FSW), people who inject drugs (PWID), men who have sex with men (MSM), and migrant workers. Because there was concern among PRs about the sustainability of the program beyond the 5 years of the grant support, the Health Intervention and Technology Assessment Programme (HITAP) was invited by the Country Coordinating Mechanisms (CCM) to take part alongside PRs and Sub-PRs in an evaluation to assess the costs and cost-effectiveness of this ongoing program. The results of this study will be used to improve program performance and support policy decision making by the Thai government in terms of whether and how the program continues at the end of the period of Global Fund support. Using routine administrative data, program costs and outcomes in terms of population reached by CHAMPION were estimated in international dollars at I$2,333/ PWID, I$270/FSW, I$162/MSM, I$161/migrant. These estimations were much higher than the cost per person in comparable programs for PWID in Bangladesh (I $727/PWID) and for FSW in India (I$129/FSW). The higher costs per person in Thailand may be explained by the shorter duration of the program (one and a half years for CHAMPION vs. three years for the Bangladesh project, and two years for the Indian project), which may have lead to higher fixed start-up costs that made up a significant proportion of the overall costs per person (a proportion which falls significantly for longer projects). Second, and more importantly, this higher cost may be due to Thailand’s lack of a harm reduction policy and the presence of harsh criminal sanctions for PWID, which made it more difficult to recruit PWID to the CHAMPION scheme. In its conclusion, the study suggests an urgent need to improve program performance if CHAMPION is to offer value for money in the Thai setting.

HTAs are not only recommended at the pre-implementation stage, they are also effective monitoring and evaluation tools. Even though interventions may be primarily designed to take into account context-specific issues, it is essential that examinations be carried out to determine whether they work well and remain efficient in practice, particularly when implemented together with other interventions within and outside Global Fund programs. Unlike ex-ante assessments, on-going HTAs can take into account primary research and focus on context-dependent issues, e.g. willingness of target populations to participate in the program, adherence to intervention protocol by providers and end-users, or other sectors’ responses to the program (Table 1). These kinds of HTAs should also pay particular attention to the key surrogate outcome indicators identified in the ex-ante assessment. It is also possible that results from the ex-ante assessment be used as benchmarks for the assessment at this stage.

Lastly, it is advised that HTA be included as part of the final report that all CCMs submit to the Global Fund when a particular program comes to an end. This should help the Global Fund incorporate feedback mechanisms regarding the requirements, constraints, and potential of the PR and SRs, who will ultimately determine if the Global Fund will achieve its goals. Collective information on the value for money of various programs implemented in different settings (*value for money guidance*) will be a valuable resource for the Global Fund and other development agencies in making future resource allocation decisions. An equally important implication is that ex-post HTA can provide information to various responsible authorities in recipient countries to encourage them to continue financing programs that are proven to be good value for money. As sustainability is a serious concern for all parties involved in the delivery of external aid, and local governments are often in a difficult situation on whether to continue the support for initiatives previously funded by external donors, ex-post assessments can provide good opportunities to inform decision makers in recipient countries about the usefulness, value for money, and other implications the program might have if it is maintained.

### Challenges of using HTA for the Global Fund

There are many challenges to overcome if HTAs are to help the Global Fund make a shift in funding projects that have a real impact.

#### Complexity of HTAs for the Global Fund

HTAs for the Global Fund need to be transparent, robust, and adaptable to local contexts. They also need to take into account the local factors that may influence the outcomes and impacts of investment. Unfortunately, typical HTAs tend to be articulated around a single or limited number of health interventions in a context-free environment [41]. Because Global Fund programs often relate to arrangements of health system and services, and encompass multiple interventions that are packaged together, HTAs for the Global Fund must allow for multiple interventions and outcomes being evaluated at the same time. Global Fund HTAs should also take care to take into account the synergic effects of multiple intervention interactions on population health as well as on particular disease burdens. For example, HTAs could be used to assess the synergic effects of incorporating maternal and child health activities into Global Fund programs for HIV (which is an approach that has been recently signed off by the Global Fund Board).

Moreover, HTA should provide not only value for money information but also social, institutional and ethical implications including equity issues since different societies may have different social values toward health investment. For example, although expanding antiretroviral treatment to those eligible HIV patients but not on it would prove to be much more costly than investing in second or third line treatments for those failed from the first-line regime, decision makers in particular settings may opt to support a program to reach out the marginal groups due to equity consideration.

#### HTA facilities for the Global Fund

Since the Global Fund clearly states in its mission that it is a financing mechanism rather than an implementing institution, it is essential that the Global Fund maintain its role in promoting financial accountability and not develop its in-house HTA capacity. Indeed, not only would this go beyond the fund’s specific remit, it would also create a conflict of interests in terms of eroding the separation between purchaser and provider. However, HTAs can be expensive. A review of HTA agencies found that the average cost per health technology assessment in ten different countries ranges from $3,000 to $650,000 [24]. As a result, independent contracting for the provision of technical support for HTAs might be effectively mobilized through the creation of global and/or regional HTA facilities. These HTA facilities would house the HTA research team, reducing costs through economies of scale, and would have the capacity to provide technical support to local staff in LICs and MICs as part of the Global Fund’s capacity building and health system strengthening strategy. A global HTA facility could also be put in place to accredit regional and national HTA facilities to undertake HTA pertinent to a Global Fund program, while also serving as a hub for the collection of HTA-related information and advancing HTA methods for complex interventions (The Value for Money working group, 2013, [42-44]).

#### Increased investment in CCMs

Although implementation capacity is one factor that determines a country’s readiness for funding, evidence demonstrates that CCMs only used about 1% of the Global Fund expenditure for administrative costs at their headquarters in 2009 [45]. A 2008 paper from the Center for Global Development notes that Local Fund Agents, tasked with overseeing CCMs, lack the expertise and capacity for program monitoring [46]. This lack of funding may be a limiting factor for thorough reviews and the incorporation of HTA into grant design and proposals. This warrants improved investment in CCMs and their partners to conduct monitoring and evaluations. Increasing the placement of HTA experts in CCMs and PRs, such as in ministries of health or ministries of finance, should be considered because these experts would bring HTA knowledge and insights to the country level and ensure the incorporation of cost-effectiveness into all steps of grant application and implementation.

#### Incentives for HTA

The Global Fund has enjoyed reputational benefits due to its promotion of performance-based financing. It is possible that the use of HTAs may help this reputation to grow. Firstly, the Global Fund would be able to make better-informed interventions, due to the evidence garnered by HTAs and by complying with the HTA-informed *value for money guidance*. The Global Fund is one of the largest suppliers of antiretroviral drugs in the world—as well a primary financier of other commodities, including ITNs [36]. Health technologies and medicines consume a significant portion of funds; currently almost 40% of Global Fund Grants are used for the procurement and management of pharmaceuticals and health technologies [47]. Under the current structure of the Global Fund, the interventions funded are selected by the PR and CCM during the proposal design process. There is no evidence that the Global Fund in any way limits the choice of interventions for which applicant countries may apply. For example, there are 92 antiretroviral drugs on the list in different forms and dosages, for a total of 309 unique items, subject to the Global Fund Quality Assurance Policy. There are 98 approved products for tuberculosis and 29 for malaria. Together, this set of over 430 possible options is not exhaustive and PR may purchase other items as long as the PR can determine that it would be compliant with the quality assurance standards [48]. Clearly, *the value for money guidance* issued on the back of an HTA can be a useful tool that PRs and CCMs can use to avoid investment in high-cost and low-impact options. In addition, *the value for money guidance* can be a resource for the Global Fund (at the global level) and PRs (at the local level) to negotiate prices with those in the industry, by using its evidence as their guidance.

Alternatively, efforts can also be made for improving the efficiency of performance-based payments, which currently rely on many input indicators rather than outcomes or impacts. The use of HTA data would allow the fund to set standard payments per unit of output (e.g. number of condoms distributed) or outcome (e.g. % reduction of unsafe sex) which are closely linked to the final goals of the program (e.g. rate of HIV infection averted). This ceiling on standard payments would serve to drive substantial efficiency gains across the Global Fund’s investment portfolio and exert pressure on other funders to decrease their own unit costs and improve efficiency. Countries that cannot meet the low-end unit costs set by the Global Fund would make up the difference from other sources, leading to enhanced cost sharing. With this option, the Global Fund can focus on providing effective coverage of proven interventions, an area where current monitoring and evaluation had identified significant shortfalls.

## Conclusions

There is currently a new emphasis at the Global Fund and other global health initiatives to focus on ensuring the effective use of resources and on generating improved value for money. This timely report proposes that additional mechanisms, such as conducting HTAs before, during, and after grant implementation, can help improve the efficiency of Global Fund investment. Although some technical and management challenges merit further investigation, the costs of delaying the use of HTA evidence-informed investment in the Global Fund are high given the severe disproportion between the current resources available and the need for prevention and control of three major disease burdens worldwide.

## Competing interests

With regard to ethical approval, this was not required because of the nature of our paper. Also, the authors have no conflict of interest to declare.

## Authors’ contributions

We confirm that all named authors meet the criteria of authorship. All authors equally contributed to the course of the review and provided critical comments to the manuscript.
